# Comparison of pregnancy outcome after letrozole versus clomiphene treatment for mild ovarian stimulation protocol in poor responders

**Published:** 2014-11

**Authors:** Maryam Eftekhar, Farnaz Mohammadian, Robab Davar, Soheila Pourmasumi

**Affiliations:** 1 *Research and Clinical Center for Infertility, Shahid Sadoughi University of Medical Sciences, Yazd, Iran.*; 2 *Department of Obstetrics and Gynecology, Zanjan University of Medical Sciences, Zanjan, Iran.*

**Keywords:** *Clomiphene*, *Letrozole*, *Mild ovarian stimulation protocol*, *Poor responders*

## Abstract

**Background:**

Poor ovarian response to controlled ovarian stimulation is one of the most important interest points in assisted reproduction. Mild ovarian stimulation seems to be preferable to high dose of FSH regimens in women with a history of poor ovarian response in previous protocol. Clomiphene citrate and letrozole alone or in combination with FSH have been used in mild ovarian stimulation protocol.

**Objective:**

To compare the efficacy of letrozole and clomiphene citrate for mild ovarian stimulation on assisted reproductive technology outcomes in poor responders.

**Materials and Methods:**

In a randomized control study, 184 women aged between 20 and 45 years with the history of poor response to ovarian stimulation who were candidate for ART were randomly subdivided into two groups: group I (n= 80), women who underwent the clomiphene/gonadotropin/antagonist protocol; and group II (n= 87), patients who underwent the letrozole/gonadotropin/antagonist protocol. Groups were compared regarding implantation, chemical and clinical pregnancy rates.

**Results:**

There was a significant difference in the mean endometrial thickness between two groups (9.16±1.2 mm vs. 8.3±0.3 mm). The implantation rate was significantly higher in letrozole group compare to clomiphene group (7.2 vs. 6.6%, p=0.024 respectively). No significant differences were found in chemical and clinical pregnancy rate between two groups.

**Conclusion:**

In mild ovarian stimulation protocol, letrozole and clomiphene have similar value for the poor responder. The optimal treatment strategy for these patients remains debated.

## Introduction

Poor ovarian response to controlled ovarian stimulation (COS) is still one of the most important interest points in assisted reproduction. Poor ovarian response to gonadotropins is clearly associated with decreased ovarian reserve and advanced maternal age that has a direct and significant effect on the success of assisted reproductive technologies (ART) cycles. Choosing an optimal strategy for the women with poor response to ovarian stimulation in ART cycles still remains controversial issue (1-4).

It has been shown that administration of high doses of gonadotropins has no beneficial effect on ovarian reserves for poor-responder patients on ART treatment. While increasing the dose of follicle-stimulating hormone (FSH) may decrease the rate of cycle cancellations, but it can decline the possibility of clinical pregnancy rate and enhance the risk of spontaneous abortion (5). High doses of FSH may recruit "resistant" follicles and rescue them from atresia. Nonetheless, the retrieved oocytes are of poor quality and do not result in the generation of good quality embryos (5). Therefore, mild ovarian stimulation seems to be preferable to high dose of FSH regimens in women with a history of poor ovarian response in previous protocol (6).

Clomiphene citrate and letrozole alone or in combination with FSH have been used in mild ovarian stimulation protocol (2, 6-8). Clomiphene citrate (cc) is a non-steroidal selective estrogen receptor modulator, which acts primarily by binding with estrogen receptors at the hypothalamus. This competitive inhibition results in a perceived drop of circulating estrogen to the hypothalamus, eventually leading to increased gonadotropin secretion (5, 8, 9).

Letrozole is a potent no steroidal aromatase inhibitor that has been used as a new approach to improve ovarian stimulation response. This agent acts to inhibit prostaglandin estradiol (E_2_) synthesis, resulting in decreased negative feedback at the pituitary and increased endogenous gonadotropins secretion (1, 5, 10, 11). The aim of present study was to compare the efficacy of letrozole and clomiphene citrate for mild ovarian stimulation on ART outcomes in poor responder women.

## Materials and methods

**Figure 1:**
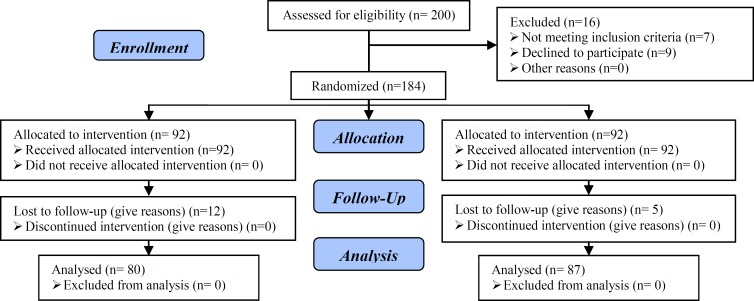
Recruitment follow-up and drop outs over the course of the study.

Two hundred women (age between 20-45 years) referring to the Research and Clinical Center for Infertility, Yazd, Iran, between March 2009 and May 2011, who had history of poor response to hyper stimulation (three or less oocytes obtained in previous cycle) were enrolled in this randomized prospective study. Sixteen patients were excluded from the study because of refusing to participate and not meeting inclusion criteria (Figure 1). In total, 184 patients were included in this study who had one or more previous failed ART cycle in which three or fewer oocytes were been retrieved and had serum E_2_ levels ≤500 pg/ml on the day of hCG administration. The exclusion criteria were as followed:

- BMI> 30 kg/m^2^
- Endocrine or metabolic disorders. (including diabetes, hypo/ hyper thyroeidism, hyper prolactinemia)
- History of ovarian surgery
- Severe endometriosis
- Azoospermia in male partner
- FSH >15 m IU/ml

The patients were divided into two groups randomly. First group consists of 92 women who received the clomiphene/ gonadotropin/ antagonist. Second groups consist of 92 patients who received letrozole/ gonadotropin/ antagonist. A computer-generated list of random numbers was used for patient classification. This study was approved by ethics committee of Research and Clinical Center for infertility, Shahid Sadoughi University of Medical Sciences, Yazd, Iran. Written informed consent was obtained from all patients before enrollment. 

**Treatment protocols**

All women received oral contraceptive for 21 days which started on the first day of previous cycle. In group I, stimulation was started by administration of clomiphene citrate (Iran hormone, Tehran, Iran) 100 mg daily from day 3 of menstruation cycle until day 7 of the cycle. Patients in group II received letrozole (Iran hormone, Tehran, Iran) from day 3 of the cycle 5mg /day for 5 days. In both groups, gonadotropins stimulation with hMG (Merional, IBSA, Lugano, Switzerland) 225-300 IU daily was started from day 5 of cycle. Patient was monitored by serial vaginal ultrasonography and measurement of serum E_2_ levels. 

As the dominant follicle reached to 14mm in mean diameter, 0.25 mg/day GnRH antagonist (Cetrotide, Sereno, Auborne, Switzerland) was started. When at least two follicles with a mean diameter of 18 mm were observed, 10000 IU hCG (Pregnyl, Organon, Netherlands) was administrated. Endometrial thickness and serum E_2_ levels were measured in the day of hCG injection. Oocyte retrieval was performed 34-36 hours after hCG injection and conventional in vitro fertilization (IVF) or intracytoplasmic sperm injection (ICSI) was done appropriately. Embryos were transferred using a Labotect catheter (Labotect, Gottingen Germany) 48-72 hours after oocytes retrieval.

Luteal phase support was started with progesterone in oil (progesterone, Aburaihan Co., Tehran, Iran) 100 mg daily intramuscularly on the day of oocyte retrieval and was continued until fetal heart activity was documented by ultrasound. The implantation rate was calculated as the ratio of the number of embryonic sacs diagnosed by sonography to the total number of the embryos transferred. Chemical pregnancy was defined by positive β-hCG, 12 days after embryos transfer. Clinical pregnancy was identified as observation of fetal heart activity by transvaginal ultrasonography that was performed three weeks after positive β-hCG. Spontaneous abortion was defined as loss of fetus with gestational age under 20 weeks.

**Statistical analysis**

Based on the pilot investigation, we assumed that 90 cases in each group is large enough to find a true difference by 80% power and 5% significance (α= 0.05, β= 0.2). The Statistical Package for the Social Sciences 15.0 software was used to analyze the data of all patients. The baseline characteristics of the two groups of patients were compared using the student *t*-test. Differences in the pregnancy outcomes of the two groups were analyzed using the Chi-square test. P≤0.05 was considered statistically significant.

## Results

Twelve patients in the clomiphene group and five women in the letrozole group were lost to follow-up. Therefore, the data of 80 women in the clomiphene group, and 87 patients in letrozole group were analyzed. The patients’ characteristics are listed in table I. Mean age; BMI, basal FSH, and duration of infertility were similar in both groups. ICSI was done on 61.7% of patients in clomiphene group, vs. 69.5% in letrozole group. 

Whilst, 38.3% and 30.5% of patients in clomiphene and letrozole group were undergoing IVF, respectively (p>0.05). There was no significant difference in the number of previous failed ART cycles, duration and total dose of hormonal stimulation, estradiol level, number of retrieved oocytes, obtained and transferred embryos between two studied groups (p>0.05). Endometrial thickness was significantly higher in letrozole group (9.16±1.24 vs. 8.39±0.38 mm; p=0.001). Implantation rate also was significantly higher in letrozole group compare to those of clomiphene group (7.2 vs. 6.6% respectively, p=0.024). 

There was no significant difference in fertilization rate, as well as chemical and clinical pregnancy rate between two groups (p>0.05) (Table II). The patients in both groups have been followed up after ET and no significant differences were found regarding miscarriage rate between clomiphene and letrozole groups.

**Table I:** Baseline characteristics of the patients in both groups

**Variable**	**Clomiphene group ** **(n=80)**	**Letrozole group ** **(n=87)**	**p-values**
Mean age (years)	37.37 ± 4.36	37.22 ± 3.95	0.807
BMI (kg/m^2^)	24.56 ± 2.53	25.2 ± 2.34	0.099
Infertility duration (years)	9.17 ± 6.53	7.93 ± 4.70	0.169
Basal FSH level (mIU/mL)	8.95 ± 4.08	8.70 ±4.20	0.705
Number of previous failed failing IVF/ICSI cycles	2.53 ± 1.12	2.16 ±0.44	0.431
Duration of hormonal stimulation (days)	11.22 ± 1.39	11.35 ± 1.23	0.520
Total number of hMG ampoules (IU)	29.60 ± 9.07	29.27 ± 9.78	0.825
E_2_ level on the day of hCG (pg/mL)	978.46 ± 614.99	1131.83 ± 677.73	0.216
Endometrial thickness (mm)	8.39 ± 0.38	9.16 ± 1.24	0.000
Number of oocyte retrieved	3.97 ± 3.18	4.25 ± 2.84	0.553
Number of embryo obtained	2.50 ± 2.04	2.31 ± 1.10	0.452
Number of embryo transferred	2.01 ± 0.92	2.00 ± 0.83	0.927

Data are presented in mean±SD.

Student’s *t* test was use.

P ≤0.05 was considered statistically significant

BMI: body mass index

FSH: follicle stimulating hormone

IVF: in vitro fertilization

ICSI: intracytoplasmic sperm injection

hMG: human menopausal gonadotropin

hCG: human chorionic gonadotropin.

**Table II:** Outcome of IVF/ICSI-ET treatment cycles in both groups

**Variable**	**Clomiphene group ** **(n=80)**	**Letrozole group ** **(n=87)**	**p-value**
Fertilization rate (%)	58.74%	62.6%	0.482
Implantation rate (%)	6.6%	7.2%	0.024
Chemical pregnancy rate a n, (%)	10.87 (11.5%)	11.80 (13.8%)	0.816
Clinical pregnancy rate b n, (%)	7 (8%)	9 (11.3%)	0.601
Miscarriage rate c n, (%)	(30%)	(27.3%)	1.00

chi-square test was use.

P ≤0.05 was considered statistically significant

a : Chemical pregnancy per cycle

b : Clinical pregnancy per cycle

c : Miscarriage rate per pregnancy

## Discussion

The patients with poor ovarian response are reported to represent about 10% of the ART population. Despite numerous developments in assisted reproduction, there is no agreement on the best stimulation protocol for poor ovarian responder patients (9). Mild ovarian stimulation is the most common protocol used in many ART centers for treatment of low responders due to lower cost and lower risk for ART procedures (12).

It has been generally accepted that clomiphene reduces uterine receptivity, and thus reduces the chances of pregnancy. The previous works reported that Treatment with clomiphene is associated with endometrial thinning in 15-50% of cases, probably due to prolonged estrogen receptor depletion in the endometrium. Compared to clomiphene, letrozole is a better alternative in terms of its effects on endometrium. On the other hand, letrozole is excreted quickly and its effect on the endometrium is reversible (8, 13-15).

In the present study, we showed that ovarian stimulation with letrozole is associated with increased endometrial thickness and higher implantation rate, when compared to clomiphene. However, there were no significant differences in chemical and clinical pregnancy rate between two groups. In agreement with our results, Mitwally and Casper reported a significantly increased endometrial thickness on the day of the hCG administration with letrozole compared with clomiphene. Although, no significant difference was noted in their study (16).

Our data revealed that miscarriage rate was similar in both groups. In contrast with our results, Al-Fozan *et al* in their randomized trial compared letrozole and clomiphene in women undergoing super ovulation and concluded that pregnancy rate in two groups was similar, but miscarriage rate was higher with clomiphene citrate (14). Badawy *et al* also, in evaluated clomiphene versus letrozole for super ovulation in women with unexplained infertility. They found no superiority between two groups. Endometrial thickness and pregnancy rate were similar in their study (17).

Karimzadeh *et al* evaluated the outcomes of micro dose and clomiphene /antagonist protocols in poor responders. They demonstrated a significantly higher gonadotropin dose and duration of stimulation in micro dose protocol. In addition, the clinical pregnancy rate was comparable between two groups in their study (9). In another work, Davar *et al* investigated ART outcomes of micro dose and letrozole/ antagonist protocols in women with low ovarian response. The researchers found that endometrial thickness, fertilization rate, and the number of embryos transferred were similar in both groups. Nonetheless, the implantation and clinical pregnancy rates were markedly higher in microdose group (2).

Yarali *et al* in a similar study compared micro dose and mild (letrozole) protocol in poor responder patients. They did not find any significant difference in the pregnancy rate between two groups (3). The use of letrozole to induce ovulation has not yet been approved by the Food and Drug Administration (FDA). Although, initial reports suggested that there may be an increased risk of congenital cardiac malformation in children born after mothers took letrozole.

Recent data demonstrated that the overall rate of malformations, including chromosomal abnormalities and congenital heart disease did not increase in children from mothers who had used letrozole to conceive (3, 18). This study result showed that mild stimulation protocol using letrozole versus clomiphene has similar effects on pregnancy rates. However, it seems that use of mild stimulation protocol in poor responders leads to reduction in the total ampoules of gonadotropin used, when compared to standard high dose protocol used for these patients without evidently compromising the pregnancy rate. 

## Conclusion

In conclusion, based on this study, letrozole and clomiphene have similar value for the poor responders in mild ovarian stimulation protocol. The optimal treatment strategy for these patients remains debated. Moreover, further large prospective randomized studies are needed to find an optimal protocol for poor responder patients.

## Note

***Registration ID in IRCT: IRCT201107146420N3***
